# Stair falls: caregiver’s “missed step” as a source of childhood fractures

**DOI:** 10.1007/s11832-014-0551-x

**Published:** 2014-01-28

**Authors:** Andrew T. Pennock, George D. Gantsoudes, Jennifer L. Forbes, Amanda M. Asaro, Scott J. Mubarak

**Affiliations:** 1 Rady Children’s Hospital San Diego, 3030 Children’s Way, Suite 410, San Diego, CA 92123 USA; 2 Department of Orthopedics, University of California, San Diego, USA; 3 Department of Orthopedics, Indiana University, Indianapolis, IN USA; 4 Physician Assistant Department, East Carolina University, Greenville, NC USA

**Keywords:** Pediatric fractures, Accidental falls, Cost analysis

## Abstract

**Background:**

The purpose of this study was to describe fractures sustained by children and to analyze the associated costs when a caretaker falls down stairs while holding a child.

**Materials and methods:**

Between 2004 and 2012, 16 children who sustained a fracture after a fall down stairs while being carried by a caregiver were identified. Parents/caregivers were interviewed to see how the fall occurred, and a cost analysis was performed.

**Results:**

The average age of the patients was 14.5 months (7–51 months). The lower extremity was involved in 15 of 16 fractures, with 8 involving the femur. The majority were buckle fractures, but all diaphyseal femur fractures were spiral. Three patients required a reduction in the operating room. All fractures healed with cast immobilization. Five patients underwent skeletal surveys, as the treating physicians were concerned about potential child abuse. The average cost of treatment was $6785 (range $948–45,876). Detailed histories from the caregivers showed that they “missed a step” due to the child being carried in front of the caregiver, obscuring their vision.

**Conclusions:**

A fall in a caregiver’s arms while going down stairs can result in multiple orthopedic injuries. The costs of treating these injuries are not insignificant, and the suspicion of child abuse can be both costly and unnecessary in the case of a true accident. While descending the stairs with a child in their arms, the caregiver should hold the child to the side so as not to obscure their vision of the step with one arm, ideally holding the handrail with the other.

**Level of evidence:**

IV case series.

## Introduction

**Fig. 1:**
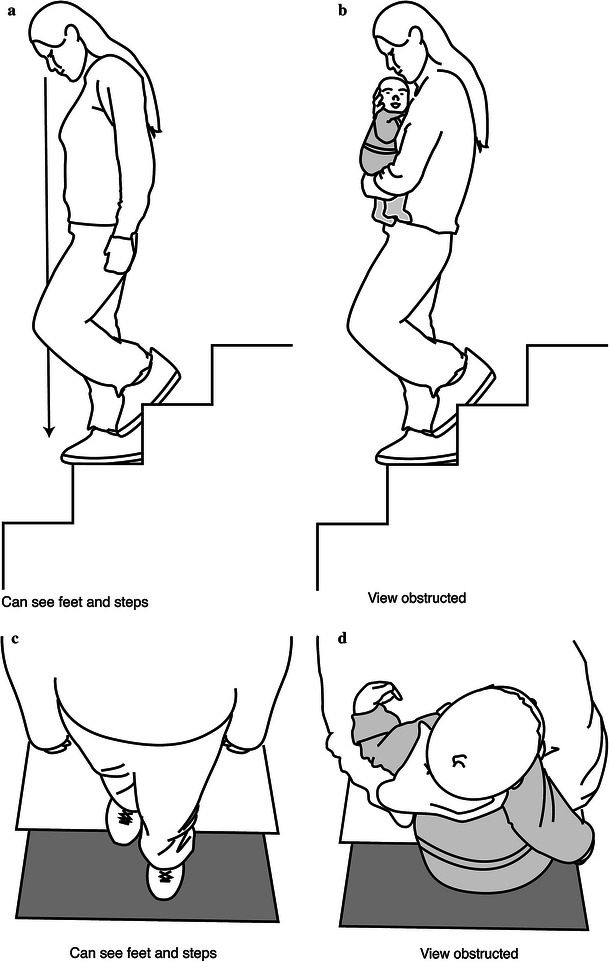
**a** Side view of the caretaker’s unobstructed view on the stairway. **b** Side view of the child obstructing the caretaker’s view. **c** Bird’s eye view of the caretaker’s unobstructed view of the stairway. **d** Bird’s eye view of how the child can obstruct the caretaker’s view on the stairway

Stairway falls are a leading cause of injury in patients less than 5 years of age [1, 2]. While the majority of these injuries occur when a child steps down the stairs, approximately 3 % of injuries will be associated with a caregiver falling while carrying a child [3]. This specific mechanism has been shown to result in injury patterns that are more likely to necessitate hospitalization and more likely to involve trauma to the head and fractures to the extremities [4]. While it is often necessary for a parent or caregiver to carry children in their arms while walking down stairs, the child may obstruct the caretaker’s view on the stairway (Fig. 1a–d). This can lead to a fall by the caretaker and an injury to the child and/or caretaker. To date, several previous studies have been conducted evaluating stairway injuries in children, but none have focused on this specific mechanism (fall from caregiver’s arms) or the injuries associated with these falls. The goal of this study was to identify injuries that may occur when a caretaker falls while carrying a child on a stairway, to understand the pathomechanics of this injury, and to perform a cost analysis of the injury.

## Materials and methods

Emergency department and orthopedic clinic records were reviewed between 2004 and 2012 to identify patients with an orthopedic injury after a fall from stairs. Sixteen children were retrospectively found to have a fracture from a “fall-in-arms” injury sustained while a caregiver was going down stairs, and were included in this study. Patient identification occurred at a routine weekly fracture conference, where every emergency department fracture in which the orthopedic service was consulted was presented. If the patient met the inclusion criteria, they were then added to the database. The following demographic and epidemiologic data were recorded for each patient: age, gender, location of injury, and mechanism of fall. Additionally, radiographs were reviewed to assess fracture location, type, displacement, and treatment. Cost analysis data was obtained from the hospital billing department and included all emergency department care, inpatient care, and subsequent follow-up. Patients were followed until discharged from the orthopedic clinics at the conclusion of treatment.

This study was granted a waiver of informed consent, including permission and assent, in accordance with 45 CFR 46.116(d) and 45 CFR 46.408, and a waiver of HIPAA authorization per 45 CFR 164.512(i). The study was authorized by the local ethical committee and was performed in accordance with the ethical standards of the 1964 Declaration of Helsinki as revised in 2000.

There are no conflicts of interest pertaining to this study. None of the authors received financial support for this study.

## Results

Sixteen children presented to the emergency room and orthopedic clinic of our hospital after sustaining an injury when their caretaker fell while carrying them down the stairs. Our billing records reveal that we, as an institution, treat approximately 9,500 fractures per year, giving an incidence of approximately 1 fracture by this mechanism per 5,000 fractures. Interviews with the parents yielded information regarding the specifics of the fall and the possible pathomechanics of the child’s injuries. The parent or caregiver noted in all cases that the child was being held in front as they descended the stairs. The child obscured their view and they missed a step and fell (Fig. 1a–d). Eight cases were female and eight were male. The age at the time of injury averaged 14 months and ranged from 7 months to 51 months of age. Fifteen of the 16 patients sustained a lower extremity injury. Eight (50 %) sustained femur fractures, six (38 %) sustained tibia fractures, and one (6 %) sustained a metatarsal fracture. There was one both bone forearm fracture (6 %); this occurred in the eldest child (51 months). All fractures were treated with a cast. Four of these patients, however, required a reduction or manipulation, three of which were performed in the operating room. All fractures healed in 4–8 weeks. No additional procedures were necessary and no complications were documented. Functionally, all patients did well, with no deficits noted at final follow-up.

Cost analysis was performed amongst our 16 patients to determine the financial burden accrued in relation to these accidental traumas (Table 1). The average total charge of the treatment of the fractures was $6,785 (standard deviation $11,183; range $948–$45,876). Five of the 16 children (31 %) had a skeletal survey, as the treating physicians were concerned about possible child abuse; the average total charge for those receiving a skeletal survey was $7,024. After a social service consultation and a skeletal survey were obtained in these cases, no patient was felt to be the victim of child abuse. For those children not requiring a skeletal survey, the average charge was $6,676. Three children (19 %) required a closed reduction of their femur fractures in the operating room; their total care charges averaged $23,568. For the children not treated in the operating room, the average charge was $2,912.

**Table 1:** Cost of fall-associated accidental traumas

Age (months)	Sex	Fractured bone(s)	Cost	Skeletal survey
18	M	Tibia/fibula	$2,607	No
7	F	Tibia	$2,024	No
12	F	Femur	$4,276	Yes
10	M	Femur	$7,861	Yes
12	M	Femur	$9,080	No
16	F	Tibia	$2,801	No
10	M	Metatarsal	$1,328	No
17	M	Femur	$19,748	Yes
12	F	Tibia/fibula	$2,594	No
7	F	Femur	$4,231	Yes
51	F	Ulna	$3,907	No
15.6 (avg. age)			$5,496 (avg. cost)	

## Discussion

The leading cause of death of children in the United States is unintentional injuries [5]. Injuries incurred on stairs, particularly in patients under the age of five, can occur with relative frequency. Three previous studies have been conducted that have evaluated stairway injuries in children [3, 4, 6]. Included in these studies were primarily children who fell while walking on the stairs, and none focused on the variable of a caretaker falling while carrying a child. It was noted, however, in these studies that children who sustained injuries while being carried tended to have more severe injuries than those who fell while walking themselves down stairs. In our study, all patients identified had incurred a fracture, and nearly all of these involved the lower extremity. Of the 16 children, half (50 %) sustained femur fractures and 38 % sustained tibia fractures. These results show that the pattern of injury differs from that of a child who falls while walking down the stairs. When a child falls alone on stairs, head and neck injuries predominate [3]. However, a child who is dropped, or fallen upon, while being carried appears more likely to sustain a long bone fracture to the lower extremity.

Pierce et al. [7] have the only series currently published that examines the incidence of femur fractures and falls down stairs. Their series evaluated femur fractures that were due to a reported fall down stairs (either solo or in a caretaker’s arms), and evaluated a plausibility model to check whether they could identify cases of nonaccidental trauma. In their subgroup of caretaker falls, this mechanism most commonly caused buckle fractures, followed by transverse/short oblique fractures. They examined the energy absorbed by the patients during falls and noted that the greater number of stairs in the fall correlated to the fracture pattern type, noting that spiral fractures were associated with falls from 1 to 3 steps and buckle fractures with falls from 4 to 15 steps. In our study, we saw an even distribution of spiral and buckle fractures that may be associated with the variability in fall heights that were observed in our patient population.

Pierce et al. [7] also employed a plausibility model to help identify if certain aspects taken from the history could be used as independent identifiers of child abuse. They found that if a caregiver could not give specific details about the fall dynamics as well as the position of the child before and after the fall, the child may have been a victim of abuse. Although this model has not been validated, and was not employed here, it may provide guidance to treating physicians and can reduce costs and avoid exposing patients to unnecessary radiation from a skeletal survey. In our series, all caregivers were able to provide a detailed history regarding the nature of their child’s injury, and subsequent non-accidental trauma work-ups suggested no cases of abuse. Additionally, no child in our study had a concomitant injury other than the fracture. This is consistent with other studies, where abused children tended to have other injuries such as bruising as well head and trunk injuries [6].

The cost of these fractures is not insignificant. The total charge for the children who received a skeletal survey as part of their work-up was $7,024. It is the duty of treating physicians—whether they are primary care physicians, emergency room physicians, or orthopedists—to be vigilant in the work-up of suspected nonaccidental trauma, and the skeletal survey is frequently the first test ordered following a history and physical.

With that said, in cases where the caregiver can provide a clear history, the skeletal survey can be deferred to minimize costs and radiation exposure to the child.

Three children in our series had to go to the operating room for a surgical reduction. All of these were for diaphyseal femur fractures that were treated with a spica cast. The costs related to the treatment of these femur fractures dwarf the costs of the other patients in this series. When taken as two separate groups, the average charge for femur fracture treatment in the emergency room was $2,912, compared to $23,568 for treatment in the operating room.

This study does have limitations and is biased toward orthopedic injuries, as all of the cases reported were obtained through orthopedic emergency room consults and clinic visits. Isolated injuries to the head or torso would not involve an orthopedic consult and thus were not included in this group. This most likely underestimates the total number of children seen at our hospital due to a fall down stairs while being carried by a caregiver. Additionally, the number of children with relatively minor injuries who did not seek medical treatment further underestimates the true incidence of this mechanism of injury.

**Fig. 2:**
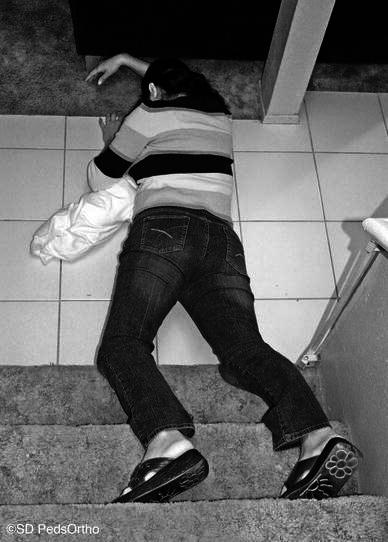
They miss the step and fall, often landing on the child as they fall

This paper describes the type of orthopedic injuries sustained during a fall down stairs while in a caregiver’s arms. What our study adds to the literature is that nearly one-third of the children underwent a skeletal survey due to concerns regarding child abuse. Additionally, the average cost of these injuries was not insignificant and averaged $6,785. Thirdly, we believe that the pathomechanics of this injury are as follows:

1. The caregiver descends the stairs carrying a child in front.
2. The size of the child obscures the view of the subsequent stair (Fig. 1a–d).
3. The caregiver misses the step and falls.
4. The momentum usually causes the caregiver and child to fall forward down the stairs (Fig. 2).

**Fig. 3:**
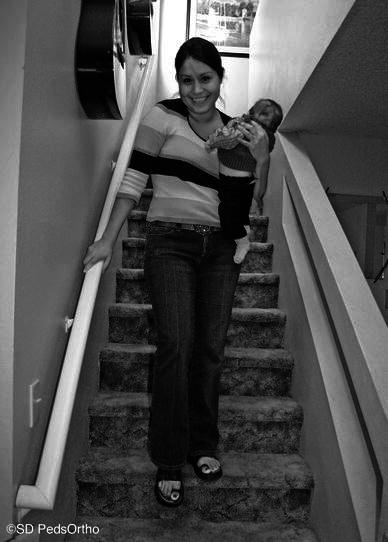
The caregiver should hold on to the handrail while traversing the steps. In addition to using the handrail, the caretaker’s view of the stairs should be unobstructed

With a better understanding of this mechanism, the treating ER physician can avoid routine skeletal surveys when the parent describes a plausible sequence, as we note above. Finally, with awareness of this mechanism, prevention of these injuries may be possible. Our recommendations are for the caregiver to hold on to the handrail while traversing the steps. In addition to using the handrail, the caretaker’s view of the stairs should be unobstructed (Fig. 3). This can be accomplished by positioning the child on the opposite side of the body to the handrail. This ensures that the caretaker has a clear view of the steps. We hope that these recommendations serve to remind healthcare providers as to the dangers of carrying children on stairways. At times this may be necessary, and can be performed safely if the proper precautions are taken.

In conclusion, a fall in a caregiver’s arms while going down stairs can result in multiple orthopedic injuries, particularly to the lower extremity. The costs of treating these injuries are not insignificant, averaging nearly $7,000, and the suspicion of child abuse can be both costly and unnecessary in the case of a true accident. While descending the stairs with a child in their arms, the caregiver should hold the child to the side so as not to obscure their vision of the step with one arm, ideally holding the handrail with the other. This simple adjustment may help minimize this potentially preventable injury.
